# Supplementary material for: A tele-health primary care rehabilitation program improves self-perceived exertion in COVID-19 survivors experiencing Post-COVID fatigue and dyspnea: A quasi-experimental study
Source: PLoS One. 2022 Aug 4;17(8):e0271802. doi: 10.1371/journal.pone.0271802 (PMC9352012; doi:10.1371/journal.pone.0271802)
Supplement: S1 File — (DOCX) [file pone.0271802.s002.docx]

***A Tele-Presential Primary Care Rehabilitation Program Improves Self-Perceived Exertion in COVID-19 Survivors experiencing Post-COVID Fatigue and Dyspnoea: A Quasi-Experimental Study***

## MAIN RESEARCHER: Mr. José Calvo Paniagua

**Center:** C.S. Arroyo de la Vega

Dirección Asistencial Norte GERENCIA ASISTENCIAL

DE ATENCIÓN PRIMARIA MADRID

Date: 03.08.2020

V.003 | 03/08/2020

# ABSTRACT

**Introduction:** Current evidence suggests that up to 70% of COVID-19 survivors develop post-COVID-19 symptoms during the months following infection. Fatigue and dyspnea appear to be the most prevalent post-COVID symptoms.

**Objectives:** To analyze the effectiveness of a telepresential Primary Health Care program of therapeutic exercises on the perceived exertion in patients with post-COVID-19 at four Primary Care Centers of the North Assistance Directorate belonging to the Assistance Management of Primary Care of the Community of Madrid.

**Design:** Quasi-experimental study.

**Participants:** COVID-19 survivors exhibiting post-COVID fatigue and dyspnea derived to four Primary Health Care centers located in Madrid.

**Intervention:** A tele-rehabilitation program based on patient education, physical activity, airway clearing, and breathing exercise interventions was structured on eighteen sessions (3 sessions/week).

**Outcomes:** Self-perceived physical exertion during daily living activities, dyspnea severity, health-related quality of life and distance walked and changes in oxygen saturation and heart rate during the 6-Minute walking test were assessed at baseline, after the program and at 1- and 3-months follow-up periods.

**Key Words:** Physiotherapy; COVID-19; Primary Care; Exercise; Physical Exertion.

## INTRODUCTION

Up to date, COVID-19 caused by SARS-CoV-2 infection affected more than 450 million cases worldwide (+180 million in Europe and +145 million in the Americas) [1]. Acute manifestations heterogeneously affect the pulmonary, cardiovascular, neurologic, hematologic and gastrointestinal systems [2]. However, recent research has focused on post-acute, long-COVID or post-COVID [3-5] since the high number of COVID-19 survivors presenting post-COVID-19 sequelae represents a major health-care challenge [6]. In fact, up to the 85% of previous hospitalized COVID-19 survivors showed post-COVID-19 symptoms during the following months after the infection [7-9].

Although multiple post-COVID symptoms have been described (e.g., memory loss, brain fog, hair loss, tachycardia, pain, skin rash, gastrointestinal problems, diarrhea, anosmia, ocular problems, ageusia) [2,5-11], fatigue and dyspnea are reported as the most common symptoms developed by this population [12]. Previous research reported fatigue and dyspnea appearance 3 months after the onset in 52-58% and 24-37% of the patients respectively [12,13]. In fact, it should be noted that only 31% of the patients did not report post-COVID fatigue or dyspnea seven months after hospital discharge [12].

This startling prevalence results in important daily living impact [14]. Evidence is consistent demonstrating the association of fatigue and dyspnea with worse quality of life and greater difficulties to perform daily living activities (i.e., walking, climbing stairs or lifting) [12,14,15]. Furthermore, since no association between pre-existing comorbidities with post-COVID-19 quality of life is observed, all these functional limitations should be specifically attributed to COVID-19 [12].

In addition to the natural course of acute COVID-19, the mandatory home isolation obeyed in Spain for more than 3 months during the outbreak in March 2020 aggravated the physical conditioning of the worldwide population at different levels. In addition to a psychological impact (i.e., increase in depressive and anxiety levels) derived from the confinement [16,17], physical deterioration implies negative metabolic changes [18] and trigger peaks in type II diabetes, both factor that could aggravate the clinical course in patients affected by COVID-19 [19].

Tele-rehabilitation programs have been widely developed during the last years (especially during the COVID-19 confinement), since is a readily accessible and feasible technology allowing long-distance communication and follow up by videoconferencing, email or texting [20]. Although currently the confinement is over, telemedicine could be considered still a feasible manner to take care of patients since this alternative clinician-patient interaction demonstrated in several disciplines to reduce the economic burden and could allow primary health care centers to reach a greater number of patients [21,22].

## OBJECTIVES AND HYPOTHESIS

**Objectives:**

Since physical activity programs reported multiple gains in physical conditioning [23], implementing a tele-rehabilitation program based on exercise may reduce the rate of aggravation and hospital admissions due to fatigue and respiratory problems, improve the patients’ quality of life and self-sufficiency and achieve fatigue and dyspnea benefits [24]. Therefore, our aim was to analyze whether a tele-presential exercise-based program developed in Primary Health Care centers improves physical exertion in post-COVID patients.

## Hypothesis:

We hypothesized that the tele-presential exercise program would significantly improve the self-perceived exertion and cardiovascular indicators in COVID-19 survivors with post-COVID fatigue and dyspnea.

## METHODS

**Study Design:**

A prospective, multicenter, single-group, quasi-experimental study.

## Setting:

Four Primary Health Care Centers located in Madrid.

## Participants:

Patients who had survived from SARS-CoV-2 infection and linked to one of the four GAAP centers participating in this study will be screened for eligibility.

## Inclusion criteria:

Individuals aged from 25 to 65 years, who had surpassed COVID-19, with a negative PCR test at the moment of the study and reporting fatigue and dyspnea as main post-COVID symptoms will be potentially eligible. Reading and signing the written informed consent will be mandatory to be included in the study.

## Exclusion criteria

Exclusion criteria include: 1) patients with other post-COVID symptoms such as gastrointestinal symptoms, anosmia, ageusia, or cognitive blurring; 2) evidence of pluri-pathology, i.e., more than two pre-existing medical comorbidities; 3, evidence of any medical co-morbidity, i.e., ischemic cardiopathy, cardiac or pulmonary insufficiency, potentially explaining fatigue or dyspnea; 4, presence of fatal medical co-morbidities such as cancer; 5, immunodeficient patients; 6, previous history of dementia or psychiatric disorders; 7, patients with severe functional limitations (Barthel index score > 90); or 8) patients with cognitive problems.

## Sample size estimation:

Sample size estimation was calculated using the G*Power v.3.1 software for Mac OS. A priori analysis to compute the required sample size setting the α, β and effect size was conducted running a mean difference statistical test for one sample cases. The input parameters were set for bilateral contrast (two-tailed), α=0.05, β=0.15 (95% power) and a moderate effect size d=0.5. These data lead to a minimum sample size of 54 participants. Due to the longitudinal nature of this study, an additional 10% sample size was included. Therefore, a sample size of 60 participants was proposed as appropriate.

**Intervention:**

The exercise-based rehabilitation program was built to be performed in a tele-presential modality by videoconference using Zoom. This program is divided in 18 sessions of 40 minutes of duration each one, three times a week (alternating days). Therefore, the total duration of the program is planned to be up to 7 weeks.

Tele-rehabilitation program chronogram.

| Session 1 | Theoretical session:   - Basic principles of anatomy and physiology - COVID-19 preventive measures (safe distance, use of facemasks, room ventilation and hand hygienization) - Sanitary education - Postural ergonomics. |
| --- | --- |
| Session 2-5 | Breathing exercises:   - Diaphragmatic breathing, costal breathing, pursed-lips breathing and airways cleaning |
| Session 6-8 | Breathing exercises  Phyisical conditioning with increasing intensity:   - Cervical, dorsal and lumbar spine active mobilizations - Lower and upper limb active mobilizations - Core training with motor control exercises |
| Session 9 | Breathing exercises  Physical conditioning with increasing intensity  Body balance training:   - Dynamic sitting control exercises - Deambulation exercises |
| Session 10-11 | Breathing exercises  Physical conditioning with increasing intensity  Functional exercises:   - Plyometric exercises   Occupational therapy exercises for daily living activities |
| Session 12-18 | Breathing exercises  Physical conditioning with increasing intensity  Functional exercises  Occupational therapy exercises  Aerobic training:   - Walking at tolerable speed |

**Outcomes:**

Outcomes will be assessed at baseline, at the end of the rehabilitation program, and one and three months after (follow-up periods).

The perceived physical exertion during their daily living activities will be assessed with the Modified Borg Dyspnea Scale (MBDS), a valid and reliable method for assessing dyspnea in patients with respiratory conditions [27].

Dyspnea severity will be classified using the modified Medical Research Council (mMRC) scale as is one of the most widely used and validated scale to assess dyspnea in daily living in chronic respiratory diseases [28].

Health-related quality of life will be assessed by using the St George’s Respiratory Questionnaire (SGRQ) [29].

The 6-Minute Walking Test (6MWT) will be performed to evaluate whether this physical demand change their heart rate, O_2_ saturation, perceived physical exertion (using again the MDBS) and distance walked [30].

## ETHICAL CONSIDERATIONS

This Project will be evaluated by the Local Ethical Committe of Gerencia Asistencial de Atención Primaria de Madrid and Hospital Universitario de la Paz.

Since participation is voluntary, participants will receive an informative document (containing the objectives of the study, eligibility criteria, what would be asked to do, how will be used their data and their right to withdraw the study) and informed consent.

## REFERENCES

1. World Health Organization. COVID-19 Dashboard, 2022. Available at: <https://covid19.who.int>
2. Zheng KI, Feng G, Liu WY, Targher G, Byrne CD, Zheng MH. Extrapulmonary complications of COVID-19: A multisystem disease? *J Med Virol*. 2021; 93(1): 323-335. doi: 10.1002/jmv.26294.
3. Fernández-de-las-Peñas C, Palacios-Ceña D, Gómez-Mayordomo V, Cuadrado ML, Florencio LL. Defining Post-COVID Symptoms (Post-Acute COVID, Long COVID, Persistent Post-COVID): An Integrative Classification. *Int J Environ Res Public Health*. 2021;18(5):2621. doi:10.3390/ijerph18052621
4. Fernández-de-las-Peñas C, Varol U, Fuensalida-Novo S, Plaza-Canteli S, Valera-Calero JA. Is the number of long-term post-COVID symptoms relevant in hospitalized COVID-19 survivors? *Eur J Intern Med*. 2022; S0953-6205(22) 00069-3. doi:10.1016/j.ejim.2022.02.013
5. Fernández-de-las-Peñas C. Long COVID: current definition. *Infection*. 2022; 50: 285-286.
6. Sykes DL, Holdsworth L, Jawad N, Gunasekera P, Morice AH, Crooks MG. Post-COVID-19 symptom burden: What is long-COVID and how should we manage it?. *Lung*. 2021;199(2):113-119. doi:10.1007/s00408-021-00423-z
7. Fernández-de-las-Peñas C, Palacios-Ceña D, Gómez-Mayordomo V, et al. Prevalence of post-COVID-19 symptoms in hospitalized and non-hospitalized COVID-19 survivors: A systematic review and meta-analysis. *Eur J Intern Med*. 2021;92:55-70. doi:10.1016/j.ejim.2021.06.009
8. Lopez-Leon S, Wegman-Ostrosky T, Perelman C, Sepulveda R, Rebolledo PA, Cuapio A, Villapol S. More than 50 Long-term effects of COVID-19: a systematic review and meta-analysis. *Sci Rep* 2021; 11(1): 16144. doi: 10.1038/s41598-021-95565-8.
9. Han Q, Zheng B, Daines L, Sheikh A. Long-term sequelae of COVID-19: A systematic review and meta-analysis of one-year follow-up studies on post-COVID symptoms. *Pathogens.* 2022; 11(2): 269. doi: 10.3390/pathogens11020269
10. Davido B, Seang S, Tubiana R, de Truchis P. Post-COVID-19 chronic symptoms: a postinfectious entity?. *Clin Microbiol Infect*. 2020;26(11):1448-1449. doi:10.1016/j.cmi.2020.07.028
11. Oronsky B, Larson C, Hammond TC, et al. A review of persistent post-COVID Syndrome (PPCS) *Clin Rev Allergy Immunol*. 2021;1-9. doi:10.1007/s12016-021-08848-3
12. Fernández-de-las-Peñas C, Palacios-Ceña D, Gómez-Mayordomo V et al. Fatigue and dyspnoea as main persistent post-COVID-19 symptoms in previously hospitalized patients: Related functional limitations and disability. *Respiration*. 2022;101(2):132-141. doi:10.1159/000518854
13. Cortés-Telles A, López-Romero S, Figueroa-Hurtado E, et al. Pulmonary function and functional capacity in COVID-19 survivors with persistent dyspnoea. *Respir Physiol Neurobiol*. 2021;288:103644. doi:10.1016/j.resp.2021.103644
14. Garrigues E, Janvier P, Kherabi Y, et al. Post-discharge persistent symptoms and health-related quality of life after hospitalization for COVID-19. *J Infect*. 2020;81(6):e4-e6. doi:10.1016/j.jinf.2020.08.029
15. Jacobs LG, Gourna Paleoudis E, Lesky-Di Bari D, et al. Persistence of symptoms and quality of life at 35 days after hospitalization for COVID-19 infection. *PLoS One*. 2020;15(12):e0243882. doi:10.1371/journal.pone.0243882
16. Ammar A, Mueller P, Trabelsi K, et al. Psychological consequences of COVID-19 home confinement: The ECLB-COVID19 multicenter study. *PLoS One*. 2020;15(11):e0240204. doi:10.1371/journal.pone.0240204
17. Sepúlveda-Loyola W, Rodríguez-Sánchez I, Pérez-Rodríguez P, et al. Impact of Social Isolation Due to COVID-19 on Health in Older People: Mental and Physical Effects and Recommendations. *J Nutr Health Aging*. 2020;24(9):938-947. doi:10.1007/s12603-020-1469-2
18. Bowden Davies KA, Pickles S, Sprung VS, et al. Reduced physical activity in young and older adults: metabolic and musculoskeletal implications. *Ther Adv Endocrinol Metab*. 2019;10:2042018819888824. doi:10.1177/2042018819888824
19. Lima-Martínez MM, Carrera Boada C, Madera-Silva MD, Marín W, Contreras M. COVID-19 and diabetes: A bidirectional relationship. *Clin Investig Arterioscler*. 2021;33(3):151-157. doi:10.1016/j.arteri.2020.10.001
20. Peretti A, Amenta F, Tayebati SK, Nittari G, Mahdi SS. Telerehabilitation: Review of the State-of-the-Art and Areas of Application. *JMIR Rehabil Assist Technol*. 2017;4(2):e7. Published 2017 Jul 21. doi:10.2196/rehab.7511
21. Fiani B, Siddiqi I, Lee SC, Dhillon L. Telerehabilitation: Development, application, and need for increased usage in the COVID-19 Era for patients with spinal pathology. *Cureus*. 2020;12(9):e10563. Published 2020 Sep 21. doi:10.7759/cureus.10563
22. Rogante M, Grigioni M, Cordella D, Giacomozzi C. Ten years of telerehabilitation: A literature overview of technologies and clinical applications. *NeuroRehabilitation*. 2010;27(4):287-304. doi:10.3233/NRE-2010-0612
23. Pinto AJ, Dunstan DW, Owen N, Bonfá E, Gualano B. Combating physical inactivity during the COVID-19 pandemic. *Nat Rev Rheumatol*. 2020;16(7):347-348. doi:10.1038/s41584-020-0427-z
24. Turolla A, Rossettini G, Viceconti A, Palese A, Geri T. Musculoskeletal physical therapy during the COVID-19 Pandemic: Is telerehabilitation the answer?. *Phys Ther*. 2020;100(8):1260-1264. doi:10.1093/ptj/pzaa093
25. Vallvé C, Artés M, Cobo E; TREND group. Estudios de intervención no aleatorizados (TREND) [Non-randomized evaluation studies (TREND)]. Med Clin (Barc). 2005;125 Suppl 1:38-42. doi:10.1016/s0025-7753(05)72208-9
26. Simera I, Moher D, Hoey J, Schulz KF, Altman DG. A catalogue of reporting guidelines for health research. Eur J Clin Invest. 2010;40(1):35-53. doi:10.1111/j.1365-2362.2009.02234.x
27. Kendrick KR, Baxi SC, Smith RM. Usefulness of the modified 0-10 Borg scale in assessing the degree of dyspnea in patients with COPD and asthma. *J Emerg Nurs*. 2000;26(3):216-222. doi:10.1016/s0099-1767(00)90093-x
28. Hsu KY, Lin JR, Lin MS, Chen W, Chen YJ, Yan YH. The modified Medical Research Council dyspnoea scale is a good indicator of health-related quality of life in patients with chronic obstructive pulmonary disease. *Singapore Med J*. 2013;54(6):321-327. doi:10.11622/smedj.2013125
29. Bohannon RW, Crouch R. Minimal clinically important difference for change in 6-minute walk test distance of adults with pathology: a systematic review. *J Eval Clin Pract*. 2017;23(2):377-381. doi:10.1111/jep.12629
30. Wang TJ, Chau B, Lui M, Lam GT, Lin N, Humbert S. Physical medicine and rehabilitation and pulmonary rehabilitation for COVID-19. *Am J Phys Med Rehabil*. 2020;99(9):769-774. doi:10.1097/PHM.0000000000001505

**INFORMATION FOR PARTICIPANTS**

**Title:** “*A Tele-Presential Primary Care Rehabilitation Program Improves Self-Perceived Exertion in COVID-19 Survivors experiencing Post-COVID Fatigue and Dyspnoea: A Quasi-Experimental Study”*

## Responsable Researcher: Mr. José Calvo Paniagua

**Physiotherapist at C.S. Arroyo de la Vega, Gerencia Asistencial Atención Primaria Madrid**

**Version 003**

**Date:**

Estimated participant,

We are contacting you to request your participation in the research project “A Tele-Presential Primary Care Rehabilitation Program Improves Self-Perceived Exertion in COVID-19 Survivors experiencing Post-COVID Fatigue and Dyspnoea: A Quasi-Experimental Study”. Our intention is that you receive the correct and sufficient information so that you can evaluate and judge whether or not you want to participate in this study. We will clarify any doubts that may arise at any time. In addition, you can consult with the people you consider appropriate.

You have the right to ask all the questions you deem appropriate and to request information, in the same way that you have the right to withdraw from the research at any time, since your participation is voluntary. The researchers can withdraw you from the project if they deem it appropriate in favor of the research.

You are invited to participate in a series of tests and questionnaires related to the evaluation of patients in the post-COVID-19 recovery phase.

Before deciding if you agree to participate in the project, it is important that you know and understand all the reasons why this research will be carried out and how the information will be used.

Due to the global epidemiological situation caused by the coronavirus and in order to prevent the spread of the virus, a series of social distancing measures are recommended, so the use of telematic healthcare can be a good tool. The objective of the study is to determine the effectiveness of a physiotherapy exercise program performed by video call/zoom for patients in the post-COVID-19 recovery phase.

You have been selected because you meet the inclusion criteria established in the project protocol after having overcome COVID-19 while in the recovery phase, and you do not present any contraindication to carry it out.

The project is planned as follows:

Potential participants who meet the criteria of the project are contacted by telephone; they are explained by telephone requesting their participation in it.

Once the potential participant accepts, they will be summoned to the C.S. Arroyo de la Vega to carry out an initial interview where all the details of the project will be explained to you, any possible doubts will be answered, you will be given this information sheet and the informed consent that you must read carefully and sign. If you meet all the requirements and there are no contraindications in the initial interview, once the consent has been signed, some tests and questionnaires will be carried out with the aim of knowing your feeling of effort and the relationship with your life, in addition, data relevant to your life will be recorded. gender, age, health or lifestyle habits.

Next, a specialized physiotherapist will begin to carry out the therapeutic exercise program with you in telepresence mode (video call).

The exercise program has a duration of 7 weeks where 3 treatment sessions per week will be carried out on alternate days with an approximate duration of 40 minutes per session.

The exercise program will be based on:

- Health education and postural ergonomics sessions, a brief introduction will be made on anatomy, physiology, known theoretical aspects of COVID-19 (hand hygiene, use of masks, social distancing), warning signs, importance of smoking cessation, weight control, nutrition, benefits of physical exercise, ergonomics and postural hygiene.

- Respiratory physiotherapy sessions, respiratory control, abdominal-diaphragmatic breathing, directed ventilation, secretion mobilization techniques, thoraco-abdominal muscle exercises.

- Physical training, optimization of the vital and pulmonary capacity of the patient. They will be carried out:

- Cervical, dorsal and lumbar spine exercises with the aim of performing active mobility and working the secondary respiratory muscles.

- Exercises for upper and lower limbs for active joint mobility and work on muscle strengthening as well as increasing tone.

- Abdominal and thoracolumbar muscle exercises to stabilize the trunk to achieve greater muscle power and balance.

- Aerobic training exercises, seeking physical improvement against effort (total re-training). They will be taught to carry out their daily activities while maintaining control of breathing and muscle fatigue. Will be performed:

- Exercises to control sitting (sitting down and getting up from a chair)

- Guided and controlled walking exercises (you will be asked for greater or lesser intensity)

- ABVDs exercises (Reproduce the movement of brushing teeth, reproduce the movement of sponge in the shower, reproduce the movement of cutting a potato, reproduce movement of cleaning windows).

- Exercises

If you have any doubt, please feel free to contact with the responsable researcher of this project:

**Mr. JOSÉ CALVO PANIAGUA**

**PHYSIOTHERAPIST**

**C.S. Arroyo de la Vega. ALCOBENDAS (GERENCIA ASISTENCIAL ATENCIÓN PRIMARIA MADRID)**

**Phone number: 00 34 696510394**

**Mail:** [**jose.calvo@salud.madrid.org**](mailto:jose.calvo@salud.madrid.org)

## INFORMED CONSENT

**Title:** “*A Tele-Presential Primary Care Rehabilitation Program Improves Self-Perceived Exertion in COVID-19 Survivors experiencing Post-COVID Fatigue and Dyspnoea: A Quasi-Experimental Study”*

## Responsable Researcher: Mr. José Calvo Paniagua

**Physiotherapist at C.S. Arroyo de la Vega, Gerencia Asistencial Atención Primaria Madrid**

**Version 003**

**Date:**

I, (name, surname and ID) ,

declare in my own representation that:

- I have read the informative document the researchers provided me, and I had the opportunity of asking all my doubts and I received all the necessary information about the study.
- Furthermore, I understand that I can withdraw the study whenever I want, giving no explanations and without bad consequences to my medical attention.
- I agree to participate in this study.

Therefore, I manifest I understood my rights and I agree to participate in this study.

Signed in Madrid, Day: ________Month: __________20 .

Signature:........................................ Signature:.........................................

Researcher:

## CONFIDENCIALITY DISCLOSURE/DATA PROTECTION

## CONSENT FOR RESEARCHING PURPOSES

By means of this document and in compliance with current regulations on data protection, I am informed and expressly consent to the processing of data from my clinical history as well as those resulting from your participation in the study “A Tele -Presential Primary Care Rehabilitation Program Improves Self-Perceived Exertion in COVID-19 Survivors experiencing Post-COVID Fatigue and Dyspnoea: A Quasi-Experimental Study”. The Data Controller is Hospital Universitario La Paz (including Hospital Carlos III-Hospital Cantoblanco) whose Data Protection Officer (DPD) is the "PDP Committee of the Ministerio de Sanidad of the Community of Madrid" addressed at Plaza Carlos Trías Bertrán nº7 (Edificio Soluble) Madrid 28020 protecciondedatos.sanidad@madrid.org. The purpose is to analyze the effectiveness of a telepresence program of therapeutic exercises on the perceived effort of Primary Care patients in the post-COVID-19 recovery phase.

The legal basis that legitimizes the treatment is your consent, as well as Law 14/2007, of July 3, on Biomedical Research and other current legislation on the matter. For this purpose, your data will be kept for the years necessary to comply with the obligations stipulated in the current applicable regulations, as well as while it is useful for the purpose for which it was obtained, and in any case, for at least five years. Access to my personal information will be restricted to the study doctor/s, their collaborators and other personnel participating in it, health authorities, the Hospital Research Ethics Committee and the promoter's monitors and auditors, who will be subject to the duty of secrecy inherent to their profession, when necessary, to verify the data and procedures of the study, but always maintaining their confidentiality in accordance with current legislation. No additional data communications will be made, except in those cases required by law.

By providing your data, you guarantee that you have read and expressly accepted the treatment thereof as indicated. You may exercise your rights of access, rectification, deletion, opposition, treatment limitation and portability, to the extent applicable, through written communication to the Data Controller, with address at Hospital Universitario La Paz, Paseo de la Castellana 261, 28046 Madrid, specifying your request, together with your DNI or equivalent document. Likewise, we inform you of the possibility of filing a claim with the Spanish Data Protection Agency (C/Jorge Juan, 6 Madrid 28001) www.agpd.es.

Madrid, Day: _____________Month: ______20 .

**Signature**

Mr./Mrs.

,

With ID number _____________________________, in my own representation

or representing Mr./Mrs. ,

With ID number. _________________________.
