# Supplementary material for: A tele-health primary care rehabilitation program improves self-perceived exertion in COVID-19 survivors experiencing Post-COVID fatigue and dyspnea: A quasi-experimental study
Source: PLoS One. 2022 Aug 4;17(8):e0271802. doi: 10.1371/journal.pone.0271802 (PMC9352012; doi:10.1371/journal.pone.0271802)
Supplement: S2 File — (DOCX) [file pone.0271802.s003.docx]

Efectividad de un programa de fisioterapia telepresencial sobre el esfuerzo percibido

en pacientes de Atención primaria en recuperación post enfermedad COVID-19

## INVESTIGADOR PRINCIPAL: D. José Calvo Paniagua

**Centro:** C.S. Arroyo de la Vega

Dirección Asistencial Norte GERENCIA ASISTENCIAL

DE ATENCIÓN PRIMARIA MADRID

Fecha: 03 de Agosto de 2020

VERSIÓN 003 | 03/08/2020

# RESUMEN

**Introducción:** La fisioterapia en Atención Primaria tiene una función princi- pal en la prevención de la enfermedad y promoción de la salud, sin embargo, desconocemos la intervención de la fisioterapia de Atención Primaria en la recuperación de los pacientes que han sufrido infección por COVID-19.

**Objetivos:** Analizar la efectividad de un programa telepresencial de ejerci- cios terapéuticos sobre el esfuerzo percibido de pacientes de Atención Pri- maria en fase de recuperación post COVID-19 de cuatro Centros de Atención Primaria de la Dirección Asistencial Norte pertenecientes a la Gerencia Asis- tencial de Atención Primaria de la Comunidad de Madrid.

**Diseño:** Estudio cuasi-experimental pre-post con un solo grupo.

**Sujetos de estudio:** Pacientes con resultado positivo en infección por CO- VID-19 mediante PCR, que tengan resultado NEGATIVO en el momento del estudio y que se encuentren en fase de recuperación. Se seleccionarán en cuatro Centros de Atención Primaria. Se necesitan 71 pacientes (teniendo en cuenta el número de pérdidas) para determinar la efectividad del programa con un intervalo de confianza del 95% y una potencia del 80%.

Pacientes con posibilidad de realizar tratamiento en modalidad telepresencial.

**Intervención:** Los participantes seleccionados que cumplan los criterios de inclusión firmarán el consentimiento informado y realizarán un programa de ejercicios terapéuticos en la modalidad telepresencial. El programa estará ba- sado en educación sanitaria, ejercicios respiratorios, ejercicios de entrena- miento físico y ejercicios aeróbicos. Se realizarán un total de 18 sesiones alternas 3 días a la semana en modalidad telepresencial y se realizarán tres mediciones a cada uno de los participantes.

**Variables:** Puntuación disnea del British Medical Research Council, Cuestionario de calidad de vida St. George y test de marcha 6 minutos.

**Palabras clave:** Fisioterapia, COVID-19, Atención Primaria, Ejercicio, Esfuerzo físico

## INTRODUCCIÓN

Hasta la fecha, el COVID-19 causado por la infección por SARS-CoV-2 afectó a más de 450 millones de casos en todo el mundo (+180 millones en Europa y +145 millones en América) [1]. Las manifestaciones agudas afectan heterogéneamente los sistemas pulmonar, cardiovascular, neurológico, hematológico y gastrointestinal [2]. Sin embargo, investigaciones recientes se han centrado en post-COVID, post-COVID prolongados o post-agudos [3-5] ya que la gran cantidad de sobrevivientes de COVID-19 que presentan secuelas post-COVID-19 representa un desafío importante para la atención de la salud [6]. De hecho, hasta el 85% de los sobrevivientes de COVID-19 hospitalizados anteriores mostraron síntomas post-COVID-19 durante los meses posteriores a la infección [7-9].

Aunque se han descrito múltiples síntomas post-COVID (por ejemplo, pérdida de memoria, confusión mental, pérdida de cabello, taquicardia, dolor, erupción cutánea, problemas gastrointestinales, diarrea, anosmia, problemas oculares, ageusia) [2,5-11], fatiga y disnea se reportan como los síntomas más comunes desarrollados por esta población [12]. Investigaciones previas reportaron aparición de fatiga y disnea 3 meses después del inicio en 52-58% y 24-37% de los pacientes respectivamente [12,13]. De hecho, cabe señalar que solo el 31% de los pacientes no reportaron fatiga o disnea post-COVID siete meses después del alta hospitalaria [12].

Esta sorprendente prevalencia da como resultado un importante impacto en la vida diaria [14]. La evidencia es consistente al demostrar la asociación de la fatiga y la disnea con una peor calidad de vida y mayores dificultades para realizar actividades de la vida diaria (es decir, caminar, subir escaleras o levantar objetos) [12,14,15]. Además, dado que no se observa asociación entre las comorbilidades preexistentes con la calidad de vida post-COVID-19, todas estas limitaciones funcionales deben atribuirse específicamente a COVID-19 [12].

Además del curso natural de la COVID-19 aguda, el aislamiento domiciliario obligatorio observado en España durante más de 3 meses durante el brote de marzo de 2020 agravó la condición física de la población mundial en diferentes niveles. Además de un impacto psicológico (es decir, aumento de los niveles depresivos y de ansiedad) derivado del confinamiento [16,17], el deterioro físico implica cambios metabólicos negativos [18] y desencadena picos en la diabetes tipo II, ambos factores que podrían agravar el cuadro clínico. curso en pacientes afectados por COVID-19 [19].

Los programas de tele-rehabilitación se han desarrollado ampliamente durante los últimos años (especialmente durante el confinamiento por COVID-19), ya que es una tecnología de fácil acceso y factible que permite la comunicación a larga distancia y el seguimiento por videoconferencia, correo electrónico o mensajes de texto [20]. Aunque actualmente el confinamiento ha terminado, la telemedicina podría considerarse todavía una forma factible de atender a los pacientes ya que esta interacción alternativa médico-paciente demostró en varias disciplinas reducir la carga económica y podría permitir que los centros de atención primaria de salud lleguen a un mayor número de pacientes [21,22].

## OBJETIVOS E HIPÓTESIS

**Objetivos:**

Dado que los programas de actividad física informaron múltiples ganancias en el acondicionamiento físico [23], la implementación de un programa de tele-rehabilitación basado en el ejercicio puede reducir la tasa de agravamiento y los ingresos hospitalarios por fatiga y problemas respiratorios, mejorar la calidad de vida y la autosuficiencia de los pacientes. y lograr beneficios de fatiga y disnea [24]. Por ello, nuestro objetivo fue analizar si un programa basado en ejercicios telepresenciales desarrollado en centros de Atención Primaria de Salud mejora el esfuerzo físico en pacientes post-COVID.

## Hipótesis:

Presumimos que el programa de ejercicio telepresencial mejoraría significativamente el esfuerzo autopercibido y los indicadores cardiovasculares en sobrevivientes de COVID-19 con fatiga y disnea post-COVID.

## METODOLOGÍA

**Diseño:**

Estudio cuasi experimental pre-post de un solo grupo, longitudinal y prospectivo.

## Ámbito de estudio:

Cuatro Centros de Atención primaria de la Dirección Asistencial Norte de la Comunidad de Madrid pertenecientes a la GAAP.

## Sujetos de estudio:

Pacientes en fase de recuperación por COVID-19 pertenecientes a cuatro centros de Atención Primaria de la Comunidad de Madrid de la Dirección Asistencial Norte pertenecientes a la GAAP con resultado positivo en infección por COVID-19 mediante PCR y que tengan resultado NEGATIVO en el momento del estudio y que se encuentren en fase de recuperación.

## Criterios de inclusión:

Serán potencialmente elegibles personas de 25 a 65 años, que hayan superado la COVID-19, con prueba PCR negativa al momento del estudio y que reporten fatiga y disnea como principales síntomas post-COVID. Será obligatorio leer y firmar el consentimiento informado por escrito para ser incluido en el estudio.

## Criterios de exclusión

Los criterios de exclusión incluyen: 1) pacientes con otros síntomas posteriores a la COVID, como síntomas gastrointestinales, anosmia, ageusia o confusión cognitiva; 2) evidencia de pluripatología, es decir, más de dos comorbilidades médicas preexistentes; 3, evidencia de cualquier comorbilidad médica, es decir, cardiopatía isquémica, insuficiencia cardíaca o pulmonar, que podría explicar fatiga o disnea; 4, presencia de comorbilidades médicas mortales como el cáncer; 5, pacientes inmunodeficientes; 6, historia previa de demencia o trastornos psiquiátricos; 7, pacientes con limitaciones funcionales graves (índice de Barthel > 90); o 8) pacientes con problemas cognitivos.

## Tamaño de la muestra:

La estimación del tamaño de la muestra se calculó utilizando el software G*Power v.3.1 para Mac OS. Se llevó a cabo un análisis a priori para calcular el tamaño de muestra requerido estableciendo alfa, beta y el tamaño del efecto ejecutando una prueba estadística de diferencia de medias para casos de una muestra. Los parámetros de entrada se establecieron para contraste bilateral (dos colas), alfa=0.05, beta=0.15 (95% de potencia) y un tamaño de efecto moderado d=0.5. Estos datos conducen a un tamaño mínimo de muestra de 54 participantes. Debido a la naturaleza longitudinal de este estudio, se incluyó un tamaño de muestra adicional del 10%. Por lo tanto, se propuso un tamaño de muestra de 60 participantes como apropiado.

**Intervención:**

El programa de rehabilitación basado en ejercicios fue construido para ser realizado en modalidad telepresencial por videoconferencia a través de Zoom. Este programa se divide en 18 sesiones de 40 minutos de duración cada una, tres veces por semana (días alternos). Por lo tanto, se prevé que la duración total del programa sea de hasta 7 semanas.

| Sesión 1 | Sesiones teóricas:   - Principios básicos de anatomía y fisiología - Medidas de prevención del COVID-19 (distancia social, uso de mascarillas, ventilación de espacios e hygiene de manos) - Educación sanitaria - Educación en ergonomía y postural |
| --- | --- |
| Sesión 2-5 | Ejercicios respiratorios:   - Respiración diafragmática, costal, respiración con labios fruncidos y limpieza de vías aéreas |
| Sesión 6-8 | Ejercicios respiratorios  Entrenamiento físico con intensidad creciente:   - Movilización activa de la región cervical, dorsal y lumbar - Movilización active de miembros - Entrenamiento del core lumboabdominal y ejercicios de control motor |
| Sesión 9 | Ejercicios respiratorios  Entrenamiento físico con intensidad creciente  Ejercicios propioceptivos:   - Ejercicios de sedestación dinámica - Deambulación |
| Sesión 10-11 | Ejercicios respiratorios  Entrenamiento físico con intensidad creciente  Ejercicios funcionales:   - Ejercicios pliométricos   Terapia ocupacional para las actividades de la vida diaria |
| Sesión 12-18 | Ejercicios respiratorios  Entrenamiento físico con intensidad creciente  Ejercicios funcionales:  Terapia ocupacional para las actividades de la vida diaria  Ejercicio aeróbico:   - Marcha a velocidad tolerable |

**Variables a estudio:**

Los resultados se evaluarán al inicio, al final del programa de rehabilitación y uno y tres meses después (períodos de seguimiento).

El esfuerzo físico percibido durante sus actividades de la vida diaria se evaluará con la Modified Borg Dyspnea Scale (MBDS), un método válido y confiable para evaluar la disnea en pacientes con afecciones respiratorias [27].

La gravedad de la disnea se clasificará utilizando la escala modificada del Medical Research Council (mMRC), ya que es una de las escalas más utilizadas y validadas para evaluar la disnea en la vida diaria en enfermedades respiratorias crónicas [28].

La calidad de vida relacionada con la salud se evaluará mediante el Cuestionario Respiratorio de St George (SGRQ) [29].

Se realizará el test de marcha de 6 minutos (6MWT) para evaluar si esta demanda física cambia su frecuencia cardíaca, la saturación de O2, el esfuerzo físico percibido (utilizando nuevamente el MDBS) y la distancia recorrida [30].

## Consideraciones éticas

Una vez seleccionados los participantes, se contactará vía telefónica informándoles del estudio, resolviendo las posibles dudas y verificando que se cumplen los criterios de inclusión; en la misma llamada se le preguntará si quiere formar parte del estudio y se le pide consentimiento.

Se les cita en la Unidad de Fisioterapia del C.S. Arroyo de la Vega para comprobar que se cumplan los criterios de inclusión y realizar una primera entrevista presencial donde se les explica con detalle el estudio, se les entrega una hoja informativa aclarando todas las dudas que puedan surgir, se les entrega el consentimiento informado y el consentimiento de confidencialidad/privacidad pidiéndoles que lo lean con detenimiento y ofreciéndoles resolver todo tipo de cuestiones que el paciente necesite aclarar.

## BIBLIOGRAFÍA

1. World Health Organization. COVID-19 Dashboard, 2022. Available at: <https://covid19.who.int>
2. Zheng KI, Feng G, Liu WY, Targher G, Byrne CD, Zheng MH. Extrapulmonary complications of COVID-19: A multisystem disease? *J Med Virol*. 2021; 93(1): 323-335. doi: 10.1002/jmv.26294.
3. Fernández-de-las-Peñas C, Palacios-Ceña D, Gómez-Mayordomo V, Cuadrado ML, Florencio LL. Defining Post-COVID Symptoms (Post-Acute COVID, Long COVID, Persistent Post-COVID): An Integrative Classification. *Int J Environ Res Public Health*. 2021;18(5):2621. doi:10.3390/ijerph18052621
4. Fernández-de-las-Peñas C, Varol U, Fuensalida-Novo S, Plaza-Canteli S, Valera-Calero JA. Is the number of long-term post-COVID symptoms relevant in hospitalized COVID-19 survivors? *Eur J Intern Med*. 2022; S0953-6205(22) 00069-3. doi:10.1016/j.ejim.2022.02.013
5. Fernández-de-las-Peñas C. Long COVID: current definition. *Infection*. 2022; 50: 285-286.
6. Sykes DL, Holdsworth L, Jawad N, Gunasekera P, Morice AH, Crooks MG. Post-COVID-19 symptom burden: What is long-COVID and how should we manage it?. *Lung*. 2021;199(2):113-119. doi:10.1007/s00408-021-00423-z
7. Fernández-de-las-Peñas C, Palacios-Ceña D, Gómez-Mayordomo V, et al. Prevalence of post-COVID-19 symptoms in hospitalized and non-hospitalized COVID-19 survivors: A systematic review and meta-analysis. *Eur J Intern Med*. 2021;92:55-70. doi:10.1016/j.ejim.2021.06.009
8. Lopez-Leon S, Wegman-Ostrosky T, Perelman C, Sepulveda R, Rebolledo PA, Cuapio A, Villapol S. More than 50 Long-term effects of COVID-19: a systematic review and meta-analysis. *Sci Rep* 2021; 11(1): 16144. doi: 10.1038/s41598-021-95565-8.
9. Han Q, Zheng B, Daines L, Sheikh A. Long-term sequelae of COVID-19: A systematic review and meta-analysis of one-year follow-up studies on post-COVID symptoms. *Pathogens.* 2022; 11(2): 269. doi: 10.3390/pathogens11020269
10. Davido B, Seang S, Tubiana R, de Truchis P. Post-COVID-19 chronic symptoms: a postinfectious entity?. *Clin Microbiol Infect*. 2020;26(11):1448-1449. doi:10.1016/j.cmi.2020.07.028
11. Oronsky B, Larson C, Hammond TC, et al. A review of persistent post-COVID Syndrome (PPCS) *Clin Rev Allergy Immunol*. 2021;1-9. doi:10.1007/s12016-021-08848-3
12. Fernández-de-las-Peñas C, Palacios-Ceña D, Gómez-Mayordomo V et al. Fatigue and dyspnoea as main persistent post-COVID-19 symptoms in previously hospitalized patients: Related functional limitations and disability. *Respiration*. 2022;101(2):132-141. doi:10.1159/000518854
13. Cortés-Telles A, López-Romero S, Figueroa-Hurtado E, et al. Pulmonary function and functional capacity in COVID-19 survivors with persistent dyspnoea. *Respir Physiol Neurobiol*. 2021;288:103644. doi:10.1016/j.resp.2021.103644
14. Garrigues E, Janvier P, Kherabi Y, et al. Post-discharge persistent symptoms and health-related quality of life after hospitalization for COVID-19. *J Infect*. 2020;81(6):e4-e6. doi:10.1016/j.jinf.2020.08.029
15. Jacobs LG, Gourna Paleoudis E, Lesky-Di Bari D, et al. Persistence of symptoms and quality of life at 35 days after hospitalization for COVID-19 infection. *PLoS One*. 2020;15(12):e0243882. doi:10.1371/journal.pone.0243882
16. Ammar A, Mueller P, Trabelsi K, et al. Psychological consequences of COVID-19 home confinement: The ECLB-COVID19 multicenter study. *PLoS One*. 2020;15(11):e0240204. doi:10.1371/journal.pone.0240204
17. Sepúlveda-Loyola W, Rodríguez-Sánchez I, Pérez-Rodríguez P, et al. Impact of Social Isolation Due to COVID-19 on Health in Older People: Mental and Physical Effects and Recommendations. *J Nutr Health Aging*. 2020;24(9):938-947. doi:10.1007/s12603-020-1469-2
18. Bowden Davies KA, Pickles S, Sprung VS, et al. Reduced physical activity in young and older adults: metabolic and musculoskeletal implications. *Ther Adv Endocrinol Metab*. 2019;10:2042018819888824. doi:10.1177/2042018819888824
19. Lima-Martínez MM, Carrera Boada C, Madera-Silva MD, Marín W, Contreras M. COVID-19 and diabetes: A bidirectional relationship. *Clin Investig Arterioscler*. 2021;33(3):151-157. doi:10.1016/j.arteri.2020.10.001
20. Peretti A, Amenta F, Tayebati SK, Nittari G, Mahdi SS. Telerehabilitation: Review of the State-of-the-Art and Areas of Application. *JMIR Rehabil Assist Technol*. 2017;4(2):e7. Published 2017 Jul 21. doi:10.2196/rehab.7511
21. Fiani B, Siddiqi I, Lee SC, Dhillon L. Telerehabilitation: Development, application, and need for increased usage in the COVID-19 Era for patients with spinal pathology. *Cureus*. 2020;12(9):e10563. Published 2020 Sep 21. doi:10.7759/cureus.10563
22. Rogante M, Grigioni M, Cordella D, Giacomozzi C. Ten years of telerehabilitation: A literature overview of technologies and clinical applications. *NeuroRehabilitation*. 2010;27(4):287-304. doi:10.3233/NRE-2010-0612
23. Pinto AJ, Dunstan DW, Owen N, Bonfá E, Gualano B. Combating physical inactivity during the COVID-19 pandemic. *Nat Rev Rheumatol*. 2020;16(7):347-348. doi:10.1038/s41584-020-0427-z
24. Turolla A, Rossettini G, Viceconti A, Palese A, Geri T. Musculoskeletal physical therapy during the COVID-19 Pandemic: Is telerehabilitation the answer?. *Phys Ther*. 2020;100(8):1260-1264. doi:10.1093/ptj/pzaa093
25. Vallvé C, Artés M, Cobo E; TREND group. Estudios de intervención no aleatorizados (TREND) [Non-randomized evaluation studies (TREND)]. Med Clin (Barc). 2005;125 Suppl 1:38-42. doi:10.1016/s0025-7753(05)72208-9
26. Simera I, Moher D, Hoey J, Schulz KF, Altman DG. A catalogue of reporting guidelines for health research. Eur J Clin Invest. 2010;40(1):35-53. doi:10.1111/j.1365-2362.2009.02234.x
27. Kendrick KR, Baxi SC, Smith RM. Usefulness of the modified 0-10 Borg scale in assessing the degree of dyspnea in patients with COPD and asthma. *J Emerg Nurs*. 2000;26(3):216-222. doi:10.1016/s0099-1767(00)90093-x
28. Hsu KY, Lin JR, Lin MS, Chen W, Chen YJ, Yan YH. The modified Medical Research Council dyspnoea scale is a good indicator of health-related quality of life in patients with chronic obstructive pulmonary disease. *Singapore Med J*. 2013;54(6):321-327. doi:10.11622/smedj.2013125
29. Bohannon RW, Crouch R. Minimal clinically important difference for change in 6-minute walk test distance of adults with pathology: a systematic review. *J Eval Clin Pract*. 2017;23(2):377-381. doi:10.1111/jep.12629
30. Wang TJ, Chau B, Lui M, Lam GT, Lin N, Humbert S. Physical medicine and rehabilitation and pulmonary rehabilitation for COVID-19. *Am J Phys Med Rehabil*. 2020;99(9):769-774. doi:10.1097/PHM.0000000000001505

**HOJA INFORMATIVA PARA EL PARTICIPANTE**

**Título:** “*Efectividad de un programa de fisioterapia telepresencial sobre el esfuerzo percibido en pacientes de Atención primaria en recuperación post enfermedad COVID-19.”*

## Investigador Principal: D. José Calvo Paniagua

**Fisioterapeuta C.S. Arroyo de le Vega Dirección Asistencial Norte**

## Gerencia Asistencial Atención Primaria Madrid Nº de versión 002 y fecha 08/06/2020

Estimado señor/a, nos ponemos en contacto con usted para solicitar su participación en el proyecto de investigación “*Efectividad de un programa de fisioterapia telepresencial sobre el esfuerzo percibido en pacientes de Atención primaria en recuperación post enfermedad COVID- 19.”* Nuestra intención es que reciba la información correcta y suficiente para que pueda eva- luar y juzgar si quiere o no participar en este estudio. Nosotros le aclararemos las dudas que puedan surgir en cualquier momento. Además, puede consultar con las personas que consi- dere oportuno.

Tiene derecho a realizar todas las preguntas que estime convenientes y a pedir información, del mismo modo que tiene derecho a retirarse de la investigación en cualquier momento, ya que su participación es voluntaria. Los investigadores pueden retirarle del proyecto si lo consi- deran oportuno a favor de la investigación.

Se le invita a participar en la realización de una serie de test y cuestionarios relacionados con la evaluación de los pacientes en fase de recuperación post COVID-19.

Antes de decidir si acepta participar en el proyecto, es importante que conozca y comprenda todos los motivos por los cuales se va a llevar a cabo esta investigación y cómo se va a utilizar la información.

Debido a la situación epidemiológica mundial provocada por el coronavirus y con el propósito de evitar la expansión del virus se recomiendan una serie de medidas de distanciamiento so- cial por lo que la utilización de asistencia sanitaria en modalidad telemática puede ser una buena herramienta. El objetivo del estudio es conocer la efectividad de un programa de ejerci- cios de fisioterapia realizados mediante videollamada/zoom para pacientes en fase de recupe- ración post COVID-19.

Usted ha sido seleccionado porque cumple con los criterios de inclusión establecidos en el pro- tocolo del proyecto tras haber superado COVID-19 estando en fase de recuperación, y no pre- senta ninguna contraindicación para realizarlo.

**El proyecto se plantea de la siguiente forma:**

Se contacta telefónicamente con los posibles participantes que cumplan con los criterios del proyecto, se les explica telefónicamente solicitando su participación en el mismo.

Una vez el posible participante acepte, se le citará en el C.S. Arroyo de la Vega para realizar una entrevista inicial donde se le explicarán todos los detalles del proyecto, se resolverán las posibles dudas, se le entregará esta hoja informativa y el consentimiento informado que de- berá leer con detenimiento y firmarlo. Si cumple todos los requisitos y no existen contraindi- caciones en la entrevista inicial una vez firmado el consentimiento se le realizarán unos test y cuestionarios con el objetivo de conocer su sensación de esfuerzo y la relación con su vida, además se registrarán datos relevantes a su género, edad, salud o hábitos de vida.

Seguidamente, un fisioterapeuta especializado comenzará a realizar con usted el programa de ejercicios terapéuticos en modalidad telepresencial (videollamada).

El programa de ejercicios tiene una duración de 7 semanas dónde se realizarán 3 sesiones de tratamiento a la semana en días alternos con una duración aproximada por sesión de 40 minu- tos.

**El programa de ejercicios estará basado en:**

- - Sesiones de educación sanitaria y ergonomía postural, se realizará una breve introduc- ción sobre anatomía, fisiología, aspectos conocidos teóricos del COVID-19 (higiene de manos, uso de mascarillas, distanciamiento social), signos de alerta, importancia de la deshabituación tabáquica, control del peso, nutrición, beneficios del ejercicio físico, ergonomía e higiene postural.
  - Sesiones de fisioterapia respiratoria, control respiratorio, respiración abdomino-dia- fragmática, ventilación dirigida, técnicas de movilización de secreciones, ejercicios de la musculatura toraco-abdominal.
  - Entrenamiento físico, optimización de la capacidad vital y pulmonar del paciente. Se realizarán:
    - Ejercicios de columna cervical, dorsal y lumbar con el objetivo de realizar mo- vilidad activa y trabajar la musculatura respiratoria secundaria.
    - Ejercicios de miembros superiores e inferiores de movilidad articular activa y realizar trabajo de potenciación muscular además de aumentar el tono.
    - Ejercicios de musculatura abdominal y dorsolumbar estabilizadora del tronco para lograr mayor potencia muscular y equilibrio.
  - Ejercicios de entrenamiento aeróbico, se busca la mejora física frente al esfuerzo (re- entrenamiento total). Se les enseñará a realizar sus actividades diarias manteniendo control de la respiración y de la fatiga muscular. Se realizará:
    - Ejercicios para el control de la sedestación (sentarse y levantarse de una silla)
    - Ejercicios de deambulación guiada y controlada (se le pedirá mayor o menor intensidad)
    - Ejercicios de ABVDs (Reproducir el movimiento de lavado de dientes, reprodu- cir el movimiento de esponja en la ducha, reproducir el movimiento de cortar una patata, reproducir movimiento de limpiar cristales).
    - Ejercicios de saltos controlados.

Una vez transcurridas las 7 semanas de duración del estudio, se vuelve a citar en el C.S. Arroyo de la Vega a los participantes del estudio para una segunda entrevista presencial con el obje- tivo de recoger los resultados de los test y cuestionarios que se realizaron en la primera entre- vista. Se realizará una tercera y última entrevista presencial a los 30 días de la finalización del programa de ejercicios (se realizarán los mismos test y cuestionarios de la segunda entrevista) con el objetivo de conocer si los posibles efectos son o no duraderos en el tiempo.

# Posibles beneficios

Se espera conseguir una mejora de las posibles secuelas físicas y/o respiratorias post- COVID-19, sin embargo, es posible que no obtenga ningún beneficio para su salud por participar en este estudio.

# Compensación económica

Su participación en el estudio no le supondrá ningún gasto adicional ni tendrá compensación económica alguna.

Debe saber que toda la información recogida en el estudio se utilizará de forma confidencial, sin embargo, los datos de los resultados si podrán hacerse públicos en congresos, reuniones científicas o revistas, pero siempre salvaguardando la confidencialidad de los datos individua- les de los participantes. Estos procedimientos están sujetos a lo que dispone la Ley Orgánica 3/2018, del 5 de diciembre, de Protección de Datos Personales y garantía de los derechos digi- tales (LOPDGDD).

Si tuviera alguna pregunta en el futuro sobre la revelación o el uso que se pudiera hacer de sus datos médicos, si tuviera dudas, preocupaciones o quejas sobre el estudio o su participación en él, deberá contactar con:

**Responsable del Proyecto: JOSÉ CALVO PANIAGUA**

**FISIOTERAPEUTA**

**C.S. Arroyo de la Vega. ALCOBENDAS (GERENCIA ASISTENCIAL ATENCIÓN PRIMARIA MADRID)**

**Teléfono: 696510394**

**Correo electrónico:** [**jose.calvo@salud.madrid.org**](mailto:jose.calvo@salud.madrid.org)

## CONSENTIMIENTO INFORMADO DE COLABORACION EN EL PROYECTO

**Título:** “*Efectividad de un programa de fisioterapia telepresencial sobre el esfuerzo percibido en pacientes de Atención primaria en recuperación post enfermedad COVID-19.”*

# Investigador Principal: D. José Calvo Paniagua

**Fisioterapeuta C.S. Arroyo de le Vega Dirección Asistencial Norte**

# Gerencia Asistencial Atención Primaria Madrid Nº de versión 002 y fecha 08/06/2020

Consentimiento

Yo, (nombre, apellidos y DNI) ,

declaro en mi propio nombre y representación que:

- He leído la hoja de información que se me ha entregado y he podido hacer preguntas y recibido suficiente información sobre el estudio. Además, comprendo que puedo retirarme del estudio cuando quiera, sin tener que dar explicaciones y sin que esto repercuta en mis cuidados médicos.
- Al facilitar mis datos garantizo haber leído y aceptado expresamente el tratamiento de los mismos conforme a lo indicado.
- Presto libremente mi conformidad para participar en el estudio.

Por ello manifiesto que he comprendido mis derechos y voluntariamente accedo a parti- cipar en el estudio del proyecto de investigación.

Y para que así conste lo firmó en Madrid a de 20 .

Firma:........................................ Firma:.........................................

Investigador: Paciente o familiar

## CONFIDENCIALIDAD/PROTECCIÓN DE DATOS CONSENTIMIENTO PARA ESTUDIOS DE INVESTIGACIÓN

Mediante el presente escrito y en cumplimiento de la normativa vigente en mate- ria de protección de datos, quedo informado/a y consiento expresamente el tratamiento de los datos de mi historia clínica así como los resultantes de su participación en el estu- dio “*Efectividad de un programa de fisioterapia telepresencial sobre el esfuerzo perci- bido en pacientes de Atención primaria en recuperación post enfermedad COVID-19* “. El Responsable del Tratamiento es Hospital Universitario La Paz (incluido Hospital Carlos III-Hospital Cantoblanco) cuyo Delegado de Protección de Datos (DPD) es el **“***Comité PDP de la Consejería de Sanidad de la Comunidad de Madrid”* con dirección en Plaza Carlos Trías Bertrán nº7 (Edificio Soluble) Madrid 28020 protec- [ciondedatos.sanidad@madrid.org.](mailto:ciondedatos.sanidad@madrid.org) La finalidad es analizar la efectividad de un pro- grama telepresencial de ejercicios terapéuticos sobre el esfuerzo percibido de pacientes de Atención Primaria en fase de recuperación post COVID-19.

La base jurídica que legitima el tratamiento es su consentimiento, así como la *Ley 14/2007, de 3 de julio, de Investigación biomédica* y demás legislación vigente en la materia. Con esta finalidad sus datos serán conservados durante los años necesarios para cumplir con las obligaciones estipuladas en la normativa vigente aplicable, así como mientras que sea de utilidad para la finalidad para la que fue obtenida, y en cualquier caso, al menos durante cinco años. El acceso a mi información personal quedará re- stringido al médico/s del estudio, sus colaboradores y demás personal que participe en el mismo, autoridades sanitarias, Comité Ético de Investigación del Hospital y a los monitores y auditores del promotor, quienes estarán sometidos al deber de secreto inher- ente a su profesión, cuando lo precisen, para comprobar los datos y procedimientos del estudio, pero siempre manteniendo la confidencialidad de los mismos de acuerdo a la legislación vigente. No se realizarán comunicaciones adicionales de datos, salvo en aquellos casos obligados por Ley.

Al facilitar sus datos usted garantiza haber leído y aceptado expresamente el trata- miento de los mismos conforme a lo indicado. Podrá ejercer sus derechos de acceso, rec- tificación, supresión, oposición, limitación del tratamiento y portabilidad**,** en la medida que sean aplicables, a través de comunicación escrita al Responsable del Tratamiento de Datos, con domicilio en Hospital Universitario La Paz, *Paseo de la Castellana 261, 28046 Madrid*, concretando su solicitud, junto con su DNI o documento equivalente. Asimismo, le informamos de la posibilidad de presentar una reclamación ante la Agencia Española de Protección de Datos *(C/Jorge Juan, 6 Madrid 28001)* [www.agpd.es](http://www.agpd.es/).

Y para que así conste lo firmó en Madrid a de 20 .

**FIRMADO:**

D./Dña.

,

con N.I.F. , en mi propio nombre y representación,

o en nombre y representación de D./Dña. ,

con N.I.F. (se debe acreditar tal condición)
